# Supplementary material for: Graphene Oxide Composite for Selective Recognition, Capturing, Photothermal Killing of Bacteria over Mammalian Cells
Source: Polymers (Basel). 2020 May 13;12(5):1116. doi: 10.3390/polym12051116 (PMC7285330; doi:10.3390/polym12051116)
Supplement: Supplementary file 1 [file polymers-12-01116-s001.pdf]

Supplementary Materials

Graphene Oxide Composite for Selective Recognition, Capturing, Photothermal Killing of Bacteria Over Mammalian Cells

Gang Ma [1], Junjie Qi [1,*], Qifan Cui [2], Xueying Bao [2], Dong Gao [2] and Chengfen Xing [2,*]

[1] National-Local Joint Engineering Laboratory for Energy Conservation in Chemical Process Integration and Resources Utilization, School of Chemical Engineering and Technology, Hebei University of Technology, Tianjin 300131, P.R. China; magang0629@163.com (G.M.); qijunjie@hebut.edu.cn (J.Q.)

[2] Key Laboratory of Hebei Province for Molecular Biophysics, Institute of Biophysics, Hebei University of Technology, Tianjin 300401, P.R. China; cuiqifan1228@163.com (Q.C.); XueyingBao2017@163.com (X.B.); gaodong@iccas.ac.cn (D.G.); xingc@hebut.edu.cn (C.X.)

* Correspondence: xingc@hebut.edu.cn; Tel/Fax: 86-22-60435642 (C.X.); qijunjie@hebut.edu.cn (J.Q.)

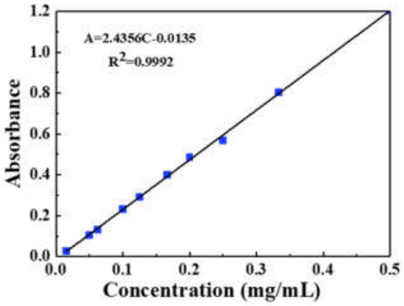

Figure S1. Standard curve of GO-PEG-NH2 in PBS.

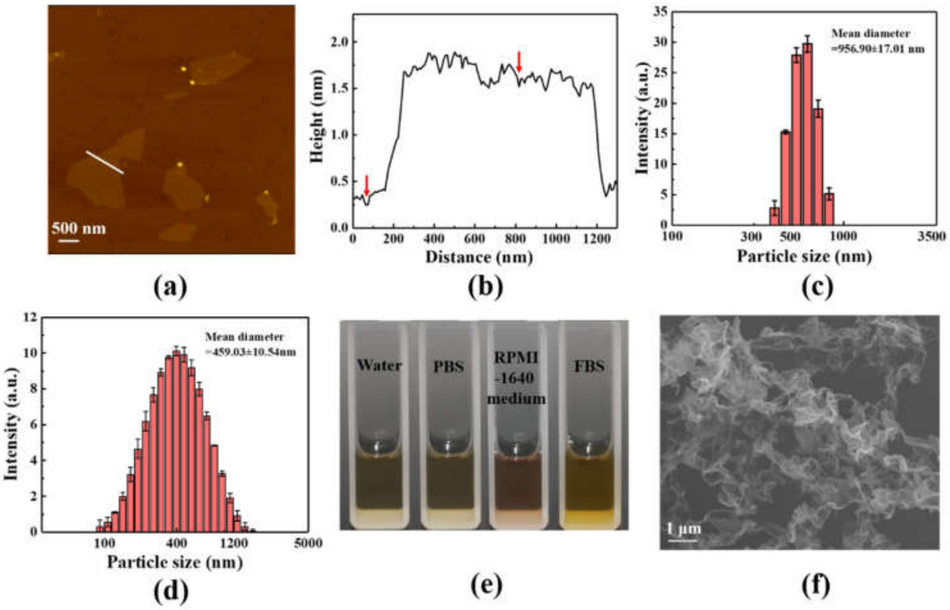

Figure S2. (**a**) AFM image of GO deposited on mica substrate. (**b**) The height profile of the AFM image. (**c**) Hydrodynamic diameter of GO measured by DLS. (**d**) Hydrodynamic diameter of GO-PEG-NH2 measured by DLS. (**e**) Photograph of GO-PEG-NH2 dispersed in different culture media for 24 h. (**f**) SEM image of GO-PEG-NH2.

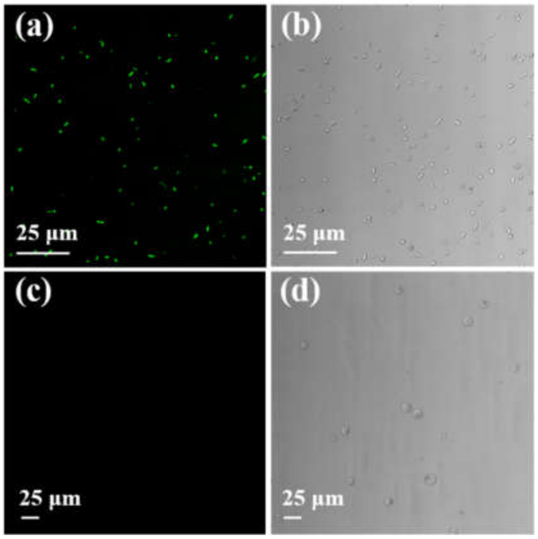

Figure S3. CLSM images of (**a, b**) *E. coli*, (**c, d**) CCRF-CEM.

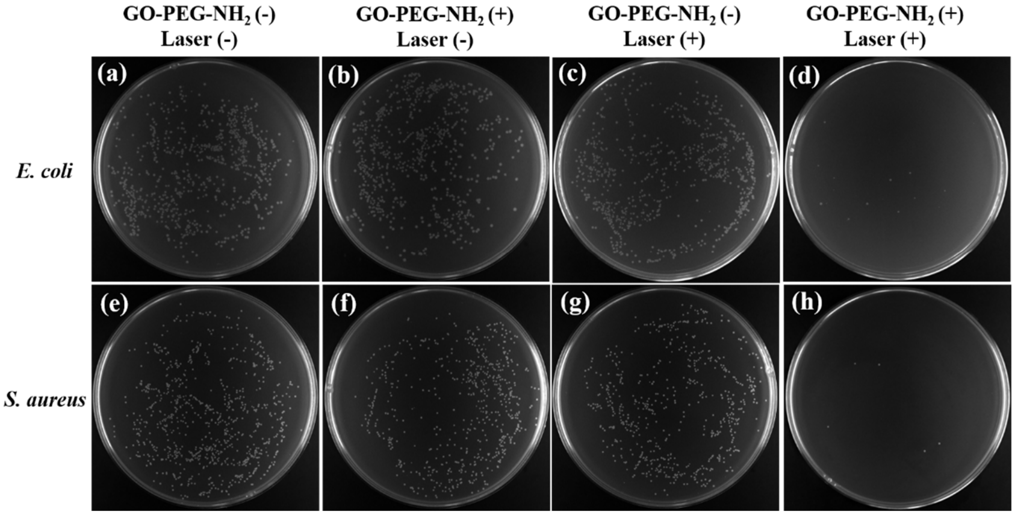

Figure S4. Gel imaging of *E. coli* and *S. aureus* colonies. (**a, e**) Plate photographs of the *E. coli* and *S. aureus* LB agar plate without GO-PEG-NH_2 under dark. (**b, f**) Plate photographs for *E. coli* and *S. aureus* LB agar plate supplemented with GO-PEG-NH_2 (50 μg/mL) under dark. (**c, g**) Plate photographs for *E. coli* and *S. aureus* LB agar plate upon 808 nm laser irradiation (1.5 W/cm^2 for 5 min). (**d, h**) Plate photographs for *E. coli* and *S. aureus* LB agar plate supplemented with GO-PEG-NH_2 upon 808 nm laser irradiation.

Table S1. Evaluation of *E. coli* colonies by plate counting method (The power density of the 808 nm laser is 1.5 W/cm^2, the irradiation time is 5 min, and the concentration of GO-PEG-NH_2 is 50 μg/mL).

Experimental Condition	Number of Colonies
Control/Non-Laser	734 ± 1
Control/Laser	676 ± 23
GO-PEG-NH_2/Non-Laser	611 ± 15
GO-PEG-NH_2/Laser	9 ± 7

Table S2. Evaluation of *S. aureus* colonies by plate counting method (The power density of the 808 nm laser is 1.5 W/cm^2, the irradiation time is 5 min, and the concentration of GO-PEG-NH_2 is 50 μg/mL).

Experimental Condition	Number of Colonies
Control/Non-Laser	776 ± 31
Control/Laser	767 ± 34
GO-PEG-NH_2/Non-Laser	714 ± 22
GO-PEG-NH_2/Laser	6 ± 5

Table S3. Evaluation of *S. aureus* and *E. coli* colonies by plate counting method (The power density of the 808 nm laser is 1.5 W/cm^2, and the irradiation time is 5 min).

Concentration (μg/mL)	Number of Colonies (*E. coli*)	Number of Colonies (*S. aureus*)
Control	734 ± 1	776 ± 31
10	258 ± 46	279 ± 47
30	69 ± 30	66 ± 35
50	9 ± 7	6 ± 5
70	3 ± 2	1 ± 1
